# Prevalence and appropriateness of psychotropic medication prescribing in a nationally representative cross-sectional survey of male and female prisoners in England

**DOI:** 10.1186/s12888-016-1055-7

**Published:** 2016-10-10

**Authors:** Lamiece Hassan, Jane Senior, Roger T. Webb, Martin Frisher, Mary P. Tully, David While, Jenny J. Shaw

**Affiliations:** 1 Faculty for Biology, Medicine and Health, The University of Manchester, Oxford Road, Manchester, M13 9PL UK; 2 School of Pharmacy, Keele University, Hornbeam Building, Keele, Staffordshire ST5 5BG UK; 3 The Manchester Pharmacy School, The University of Manchester, Oxford Road, Manchester, M13 9PL UK

**Keywords:** Prescribing, Psychotropic, Prison, Gender, Antidepressants, Antipsychotics, Mental illness

## Abstract

**Background:**

Mental illness is highly prevalent among prisoners. Although psychotropic medicines can ameliorate symptoms of mental illness, prescribers in prisons must balance clinical needs against risks to safety and security. Concerns have been raised at the large number of prisoners reportedly receiving psychotropic medicines in England. Nonetheless, unlike for the wider community, robust prescribing data are not routinely available for prisons. We investigated gender-specific patterns in the prevalence and appropriateness of psychotropic prescribing in English prisons.

**Methods:**

We studied 6052 men and 785 women in 11 prisons throughout England. This represented 7.9 % of male and 20.5 % of female prisoners nationally. Using a cross-sectional design, demographic and prescription data were collected from clinical records of all prisoners prescribed psychotropic medicines, including hypnotic, anxiolytic, antipsychotic, anti-manic, antidepressant and Central Nervous System stimulant medications. Percentages and 95 % CIs were used to estimate the prevalence of prescribing. The Prescribing Appropriate Indicators tool was used to determine appropriateness. Prevalence Ratios (PR) were generated to make age-adjusted comparisons between prisoners and the general population using a dataset supplied by the Clinical Practice Research Datalink.

**Results:**

Overall, 47.9 % (CI 44.4–51.4) of women and 16.9 % (CI 16.0–17.9) of men in prison were prescribed one or more psychotropic medicines. Compared with the general population, age-adjusted prescribing prevalence was six times higher among women (PR 5.95 CI 5.36–6.61) and four times higher among men (PR 4.02 CI 3.75–4.30). Undocumented or unapproved indications for prescriptions, not listed in the British National Formulary, were recorded in a third (34.7 %, CI 32.5–37.0) of cases, most commonly low mood and personality disorder.

**Conclusions:**

Psychotropic medicines were prescribed frequently in prisons, especially among women, and for a wider range of indications than are currently recommended. These findings raise questions about whether the prescribing of psychotropic medicines in prisons is wholly appropriate and proportionate to the level of clinical need. Prisons need to develop a wider array of treatment responses, other than medicines, to effectively tackle mental illness, challenging behaviours and distress.

**Electronic supplementary material:**

The online version of this article (doi:10.1186/s12888-016-1055-7) contains supplementary material, which is available to authorized users.

## Background

Prisoners have multiple, complex health needs, with the prevalence of mental illness greatly elevated [1, 2]. Individuals presenting with mental illness may require treatment with psychotropic medicines such as antidepressants, antipsychotics and antimanic drugs. Appropriate use of psychotropic medicines has been shown to improve symptoms and reduce relapse rates in disorders including depression [3], bipolar disorder [4, 5] and schizophrenia [6]. Furthermore, a recent study demonstrated that, among patients prescribed antipsychotics and antimanic drugs, incidence of violent crime fell during periods of medication use [7]. Nonetheless, due to the complex interplay between clinical, social and situational factors, making prescribing decisions in prison can be extremely challenging [8]. Guidance issued by the UK’s professional body for general practitioners has strongly emphasised the need to consider safety and security risks when prescribing in prisons, citing increased risks of illicit substance misuse and trading, comorbidity, self-harm and suicide [9].

It is difficult to ascertain a clear picture of psychotropic prescribing patterns in prisons. In England, robust data, with appropriate comparators, are routinely available on medicines prescribed in the community (via services such as http://www.nhsbsa.nhs.uk/3230.aspx), but prisons are not included. The Chief Inspector of Prisons has expressed concern at the large number of prisoners receiving psychotropic medicines, particularly women [10]. In spite of such concerns, the last national survey reporting on psychotropic prescribing in prisons was conducted over fifteen years ago [11]. Since then, the prison population has increased significantly, there have been major organisational changes to the delivery of prison healthcare, and new psychotropic drugs have arrived onto the market.

Studies conducted in Switzerland [12], France [13], Norway [14, 15] and the USA [16, 17] have indicated that psychotropic prescribing prevalence is elevated in prisons compared to the general population. This could simply be attributed to the higher prevalence of mental illness among prisoners. Indeed, it has been estimated that overall rates of mental illness are between 3 and 4 times higher in prison than in the wider community, although this varies for specific demographic groups and diagnoses [2]. For example, psychosis is prevalent among 3.7 % of adult prisoners compared with 0.4 % in the general population [18]. However, the relationship between psychotropic prescribing and mental illness is not entirely straightforward: whilst some patients may receive non-pharmacological treatments for mental illness, others may be prescribed psychotropic drugs ‘off label’ for reasons other than mental illness; for example, low dose amitriptyline (a tricyclic antidepressant) is sometimes used to treat neuropathic pain, but has no license for that [19]. Furthermore, whilst some studies of prescribing in prison have provided comparison data from the wider community (and other settings) [12, 14], none have adequately accounted for age and sex differences between populations. Such factors all add to the difficulty of comparing psychotropic prescribing between different settings and assessing whether prescribing in prisons is wholly proportionate to clinical need.

Building on our previous study in a single, though large, region of England [20] we conducted a national study to investigate the prevalence and appropriateness of psychotropic prescribing in English prisons to further our understanding of this important and complex area of clinical practice. Specifically, we hypothesised that psychotropic prevalence for both men and women would be significantly higher among prisoners than in the general population, after accounting for age differences. In addition, we predicted that psychotropic prescribing prevalence would be significantly higher among female than male prisoners.

## Methods

### Prisons and participants

A cross-sectional study using patient clinical records was conducted. In total, 6052 male and 785 female prisoners were surveyed from eleven prisons. Based on prison population statistics from the final week of the survey, the final sample represented approximately 7.9 % of the male and 20.5 % of the female prisoner population at that time. Two female prisons were included as well as the following male prisons: three adult local prisons, which accept convicted and unconvicted men directly from court and make up approximately half of the prison estate; three training prisons, which accept convicted and sentenced prisoners and offer rehabilitative-oriented work and education programmes; two young offender institutions, which accept 18–21 year olds; and an open prison, which have minimal security restrictions. These prisons were geographically spread across England and were recruited to represent a range of prisoner populations, including adults, young offenders (18–21 years) and both sentenced and un-convicted prisoners. No high security prisons, which hold prisoners requiring the most secure settings were available to participate in the study.

For the purposes of generating point-prevalence prescribing estimates, a ‘census day’ was selected for each participating prison, all between November 2012 and July 2013. Logistical reasons concerning data collection precluded selecting the same census day for all eleven sites, Census days were arranged on a pragmatic basis, based on when it was at convenient for the researcher to visit the prison and access clinical and prescribing records. Healthcare staff were aware that there would be a researcher visiting the department during these times.

Inclusion criteria for the study were (i) aged 18 years and over and (ii) in custody on the establishment’s census day. Approvals were obtained from an NHS research ethics committee (NHS NRES Committee North East - York, Ref: 09/H0903/54), the National Offender Management Service and each provider healthcare organisation. Approval to access clinical records without consent, in the public interest, was granted by the Ethics and Confidentiality Committee, on behalf of the National Information Board, under Section 251 of the NHS Act 2006. Patient identifiable data were accessed onsite at each individual prison only for the purpose of extracting relevant prescribing and accompanying clinical data; all data were immediately anonymised and no patient identifiable data were recorded for research purposes. Information about the study was displayed in prisons in multiple languages in advance of census days. Individuals who did not wish to participate were able to ‘opt-out’ by informing a member of healthcare staff. Those who declined were recorded and individual clinical records for these patients were not accessed as part of the research. A research advisory group of ex-prisoners met throughout the project, advising on study design, methodology and dissemination activities. The group was comprised of members who had previous experience of serving on a research user group related to mental health, suicide and self-harm, plus additional members recruited separately via personal contacts of existing members. The group included seven regular members, both men and women all of whom had experience of (a) imprisonment and (b) accessing health services in prison.

### Prison data collection

The primary outcome measure was the prevalence of psychotropic medication prescribing. Aggregate population counts for each census day were generated to provide denominators for calculating prescribing prevalence values. These estimates were stratified by age, gender, ethnicity and legal status (sentenced vs. un-sentenced). Electronic clinical information systems and/or paper records were examined to identify all patients with a current prescription for at least one psychotropic medication. Psychotropic medicines were defined as any medication listed in subchapters 4.1–4.4 of the British National Formulary (BNF) [21], including the following medicines: hypnotics and anxiolytics (chapter 4.1), which can be used to treat anxiety and sleep disorders; antipsychotics and anti-manics (4.2), used to treat psychoses, mood instability and related disorders; antidepressants (4.3), used to treat depressive and anxiety disorders; and CNS stimulants (4.4), used to treat attention deficit hyperactivity disorder. The BNF is a pharmaceutical reference book of medicines available on the NHS and is widely used by pharmacists, prescribers and other healthcare professionals for guiding the prescription, dispensing, and administration of medicines.

For each prisoner prescribed at least one psychotropic medication on the census day we extracted demographic and prescription data. Demographic data included gender, legal status, ethnicity and year of birth. We collected the following details for all psychotropic prescriptions: drug name (active ingredient), prescribed daily dose, formulation, and indication (symptoms and/or diagnosis). Additionally, we recorded the drug names of all current prescriptions for non-psychotropic medicines. Data were collected retrospectively, using information that had been prospectively recorded (at the time of clinical events, such as consultations and/or prescribing) in clinical records. Community clinical records were not available, therefore only data recorded from the time the individual entered custody were considered.

To assess prescribing appropriateness, we used the Prescribing Appropriateness Indicators (PAI), a set of standardised, validated indicators, designed specifically for use with clinical records [22]. The PAI has been validated for use in primary care populations (refs), although not prisons specifically. To streamline data collection, we discussed the nine items of the PAI and identified a subset of four items deemed most relevant to psychotropic prescribing. These were: the indication for the drug is recorded and upheld in the BNF (PAI 1); a generic (non-branded) product is prescribed if one is available (PAI 2); if a potentially hazardous drug-drug combination is prescribed, the prescriber shows knowledge of the hazard (PAI 3); and if the total daily dose is outside the range stated in the BNF, either higher or lower, the prescriber gives a valid reason (PAI 4). For each individual psychotropic prescription, we recorded whether or not each criterion was met. Some indicators in the PAI refer to indications and dose ranges listed for individual medicines listed in the BNF, based on information in manufacturers’ Summaries of Product Licences and related marketing authorisations. In addition to licensed uses of medicines, certain unlicensed uses are also listed in the BNF, although these carry the caveat of increased professional responsibility and liability for prescribers [21]. The five PAI items that were not used addressed prescribing drugs of limited value, cost, dosing frequency, treatment duration and prescribing for hypertension. We determined that the first four of these were more detailed than was necessary for the purposes of this particular study, whilst the fifth was clearly not applicable. The indicators within the PAI are designed to be applied individually rather than aggregated as a total score, therefore this approach did not affect the validity of the items we did use.

### General population comparison dataset

For comparative purposes, we used a dataset supplied by the Clinical Practice Research Datalink (CPRD) on a broadly representative national sample of patients registered with general practices in the general population. Inclusion criteria for CPRD patients were: (i) aged 18 years or over and (ii) registered with a general practice within England or Wales continuously during the eligibility period.

The CPRD supplied aggregate patient counts for (i) all patients who met the inclusion criteria, for use as denominators and (ii) all patients prescribed at least one psychotropic medicine during the eligibility period. All counts were stratified by gender and age group. Individual-level data were necessary to generate point prevalence estimates of psychotropic prescribing on a single census day, namely 30 July 2010. These data had already been obtained under the terms of a free license scheme, in advance of the prison data collection. The specific terms of the CPRD licence scheme (a 100,000 case limit) precluded us obtaining individual-level data for all those patients prescribed psychotropic medicines (*n* = 415,380); instead, we requested data for a sub-sample of 30,602 patients. This included all patients prescribed CNS stimulants (BNF subchapter 4.4) and a random sample of: 10,000 patients prescribed hypnotics and anxiolytics (4.1); 10,000 patients prescribed antipsychotic and anti-mania drugs (4.2); and 10,000 patients prescribed antidepressants (4.3). Demographic data included gender and year of birth; ethnicity, however, was incompletely recorded. Data pertaining to all medicines prescribed during the observation period were supplied, including drug name, formulation, dose, frequency, prescription date and duration.

Denominators for calculating point prevalence estimates, proportionate to the subsample for whom individual-level data were available, were generated using the following method. Individual-level data were available for 7 · 4 % (*n* = 30,602) of all patients prescribed psychotropic medicines during the eligibility period (*n* = 415,380). Given that we had individual-level data on all patients prescribed CNS stimulants, we were able to use these directly in combination with the aggregate patient counts supplied by CPRD for use as denominators to generate point-prevalence estimates of prescribing (i.e. number of patients prescribed CNS stimulants/ total number of patients = prevalence). However, given that we had only a subsample of patients prescribed psychotropic medicines in BNF subchapters 4 · 1–4 · 3, it was not possible to use the counts supplied by CPRD in a direct manner to generate point prevalence estimates for psychotropic medicines other than CNS stimulants.

Thus, we required a method to generate population denominators proportionate to the sample of patients for whom we had individual-level data. If we let the total number of CPRD patients within the eligibility period be sample *a*, then among them the sample of patients prescribed at least one psychotropic medicine will be sample *b*. In turn, the random sample of patients among sample b for whom individual-level data were provided will be sample *c*.

To generate ‘proportionate population denominators’ (sample *d*), we applied the following formula to each age/sex stratum (by BNF subchapter): *a* × (*c/b*) = *d*. Sample *d* provided the denominators for the community sample in this study. The denominators derived via this process are provided in Additional file 1: Table S1.

### Statistical analysis

Analyses were performed using version 12 of Stata software for Windows [23]. We calculated point prevalence values (as percentages) for psychotropic prescribing, and their 95 % confidence intervals using the Wilson method [24], stratified by BNF chapter, for both the prison population and the general population. These values were stratified by gender throughout because of the marked heterogeneity between the female and male prisoner populations. Prevalence ratios (PRs) were generated to compare prescribing prevalence between prisoners and the general population using the ‘ir’ command in Stata, which applies asymptotic approximations to generate 95 % confidence intervals. To account for the fact that prisoners are generally younger than the general population, the PRs were indirectly standardised for age using the CPRD dataset as the standard population. For disclosure control purposes, values of cells where the value was <5 were suppressed.

Percentages and their 95 % confidence intervals were used to describe the likelihood of psychotropic prescriptions issued in prison meeting each of the PAI items. To determine the proportion of prescriptions prescribed within the range stated in the BNF (PAI 4), we calculated the prescribed daily dose and compared this to the dose stated in the BNF. Where doses for an individual medicine differed by indication (for example, amitriptyline is prescribed at lower doses for pain than as an antidepressant), the dose for the specific indication stated in the prisoner’s clinical record was used. If there was an invalid indication, or no indication, stated, the smallest minimum and largest maximum values provided across all indications for that medicine were used instead. We also identified the drugs most frequently associated with an inappropriate indication or no indication recorded, potentially hazardous drug-drug interactions and prescribed daily doses outside the range stated in the BNF. Where there were missing data, cases with missing values were excluded from analyses involving those particular variables as outcomes or predictors (listwise deletion).

## Results

The study dataset consisted of a total of 6052 men and 785 women from 11 prisons (Table 1). Based on prison population statistics during the final month of data collection (July 2013), this sample represented 7.9 % of the male and 20.5 % of the female prison population of England. The great majority of prisoners (86.3 %) were sentenced and 82.9 % were of White ethnicity.

**Table 1:** Prisoner sample characteristics

	*N*	Percent		*n*	Percent
Gender			Prison type		
Male	6052	88.5	Local	2192	32.1
Female	785	11.5	Training	2188	32.0
Legal status			Open	598	8.7
Sentenced	5898	86.3	Youth Offender	1074	15.7
Unsentenced	924	13.5	Female	785	11.5
Other^a^	15	0.2			
Age group			Ethnicity		
18–24	2012	29.4	White	5620	82.9
25–34	1981	29.0	Black	574	8.5
35–44	1387	20.3	Asian	348	5.1
45–54	877	12.8	Mixed	182	2.7
55–64	340	5.0	Other	57	0.8
65–74	177	2.6			
75+	61	0.9			
Total	6837	100			

^a^ Includes civil prisoners and detainees

**Fig. 1:**
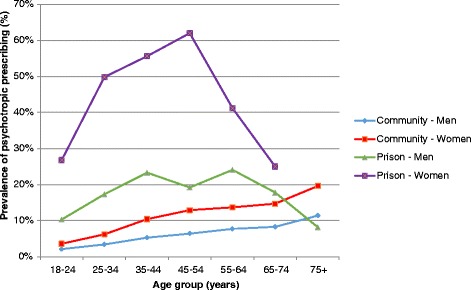
Prison and community psychotropic point-prevalence prescribing rates, by age group and sex

**Fig. 2:**
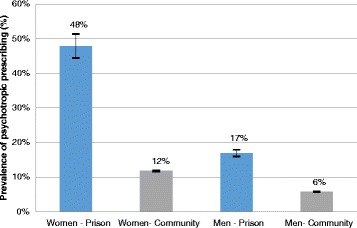
Prison and community psychotropic point-prevalence prescribing rates, by sex

Figure 1 shows the relationship between age and psychotropic prescribing, grouped by setting (prison vs. general population) and gender. Perhaps the most noteworthy feature is the strikingly high prescribing prevalence observed among female prisoners, as compared with all other groups. In prison, 47.9 % (CI 44.4–51.4) of women and 16.9 % (CI 16.0–17.9) of men were prescribed at least one psychotropic medicine (Table 2 and Fig. 2); women were almost three times more likely than men to be prescribed psychotropics (PR 2 · 7 CI 2 · 4–3 · 0). Fig. 1 indicates that psychotropic prescribing among prisoners peaked in the middle age bands (25–54 years), whilst prescribing observed in the general population appeared to show a linear increase with age. This contrast was particularly apparent for women in prison, with over half of women aged 35–54 being prescribed psychotropic medicines compared with less than 10 % of women in the general population.

**Table 2:** Crude and age-adjusted comparisons of prison sample versus general population psychotropic prescribing point prevalence estimates by gender and BNF subchapter

BNF chapter	Prison		Community (ref.)		PR (95 % CI)	
	%	*N*	%	*n*	Crude	Age adjusted
Men						
Hypnotics and anxiolytics	1.0	62	1.4	2082	0.74 (0.56–0.96)	1.27 (0.97–1.64)
Antipsychotics and antimanics	4.3	258	1.1	2430	3.67 (3.21–4.18)	4.81 (4.21–5.50)
Antidepressants	13.2	801	4.6	4183	2.87 (2.66–3.10)	4.16 (3.84–4.50)
CNS stimulants	0.7	44	0.0	457	22.57 (16.17–30.79)	12.99 (9.48–17.80)
Any	16.9	1024	5.8	6781	2.90 (2.71–3.10)	4.02 (3.75–4.30)
Women						
Hypnotics and anxiolytics	7.9	62	2.5	3756	3.11 (2.38–4.00)	7.40 (5.73–9.55)
Antipsychotics and antimanics	11.7	92	1.6	3295	7.49 (6.01–9.22)	12.74 (10.30–15.76)
Antidepressants	41.1	323	10.0	8858	4.09 (3.65–4.58)	5.55 (4.96–6.22)
CNS stimulants	0.5	<5^a^	0.0	335	22.29 (6.04–57.61)	19.01 (7.07–51.10)
Any	47.9	376	11.8	12146	4.05 (3.65–4.49)	5.95 (5.36–6.61)

*PR* Prevalence Ratio

^a^ N suppressed due to small <5 cell count

Table 2 shows that, compared with patients in the general population, rates of psychotropic prescribing in prison were 4.0 (CI 3 · 8–4 · 3) times higher among men and 6 · 0 (CI 5 · 4–6 · 6) times higher among women after adjustment for age. The most commonly prescribed psychotropic medicines in prison were antidepressants, both for men and women. In total, 1175 (67.5 %) of the 1740 separate prescriptions for psychotropic medicines identified in prison, were for antidepressants (Table 3). Collectively, the class of medicines known as selective serotonin reuptake inhibitors accounted for the greatest percentage of antidepressant prescriptions in prison (45.1 %, *n* = 530). Tricyclic antidepressants accounted for a lower proportion of antidepressant prescriptions in prison than in the community (14.9 % vs. 26.5 %; PR 0.56, 95 % CI 0.49 to 0.65). Individually, mirtazapine was the most frequently prescribed antidepressant in prison (35.2), but accounted for just 8.3 % of community antidepressant prescriptions (PR 4.26, 95 % CI 3.87 to 4.69).

**Table 3:** Top five most commonly prescribed psychotropic medicines in prison (descending order), compared with the community, by BNF subchapter and drug

	Drug	Prison %	*n*	Community %	*n*
Hypnotics and anxiolytics					
1	Diazepam	53.4	70	21.5	1376
2	Zopiclone	16.0	21	32.3	2067
3	Chlordiazepoxide	13.7	18	0.9	55
4	Promethazine	9.2	12	2.6	164
5	Melatonin	2.3	<5^a^	0.8	48
All		100	131	100	6395
Antipsychotics					
1	Olanzapine	30.9	119	14.6	935
2	Quetiapine	28.6	110	12.7	812
3	Risperidone	10.1	39	9.0	573
4	Carbamazepine	9.4	36	21.2	1355
5	Chlorpromazine	2.6	10	2.9	186
All		100	385	100	6404
Antidepressants					
1	Mirtazapine	35.2	413	8.3	1157
2	Citalopram	18.2	214	27.3	3826
3	Sertraline	13.0	153	7.4	1043
4	Fluoxetine	11.2	132	12.8	1801
5	Amitriptyline	8.8	104	16.7	2338
All		100	1175	100	14028

^a^ N suppressed due to small <5 cell count

Women were eight times more likely than men to be prescribed hypnotic and anxiolytic medicines (PR 7 · 84 CI 5 · 42–11 · 36). As Table 4 shows, diazepam accounted for half (53.4 %) of all hypnotic and anxiolytic prescriptions in prison, but only 21.5 % in the community. Prescribing rates for antipsychotics and CNS stimulants were much higher among prisoners than primary care patients in the general population (Table 2). Among antipsychotics and antimanics drugs, second generation antipsychotics were the most frequently prescribed drugs (76.1 %, *n* = 293), followed by antimanics (14.6 %, *n* = 56) and first generation antipsychotics (8.6 %, *n* = 33). Olanzapine and quetiapine, collectively accounted for over half of all prescriptions within this BNF chapter (Table 3). There were 49 instances of CNS stimulant prescribing in prison, 90.9 % (*n* = 40) of which were prescribed to treat Attention Deficit Hyperactivity Disorder (ADHD). Methylphenidate accounted for 75.5 % (*n* = 37) of prescriptions and atomoxetine accounted for the remaining 24.5 % (*n* = 12). Among men, the majority of prescriptions for CNS stimulants came from youth offender establishments (72.7 %, *n* = 32).

**Table 4:** Proportion of prison prescriptions for psychotropic medications that met each PAI indicator by gender and BNF subchapter

PAI Indicator^a,^		BNF Sub-chapter									
		Hypnotics & anxiolytics		Antipsychotics & antimanics		Antidepressants		CNS Stimulants		Any Psychotropic	
		%	*n*	%	*n*	%	*n*	%	*n*	%	*n*
Men											
1	Valid indication^b^	90.5	57	50.5	141	61.8	512	90.9	40	62.0	758
2	Generic product	100	64	97.5	273	100	832	100	44	99.4	1222
3	No drug-drug interaction	93.7	59	80.7	221	87.1	714	93.2	41	86.2	1043
4	Valid dose range	91.7	55	81.7	227	97.5	809	95.4	41	93.5	1132
Women											
1	Valid indication^b^	85.7	54	53.9	55	76.8	262	100	5	73.4	378
2	Generic product	98.5	66	99.1	104	100	343	100	5	99.6	522
3	No drug-drug interaction	100	59	69.0	69	84.4	286	100	5	83.4	423
4	Valid dose range	98.3	59	77.9	81	94.1	321	100	5	91.4	466
a Key											
PAI Indicator											
1	The indication for the drug is recorded and upheld in the BNF.										
2	A generic product is prescribed, if one is available.										
3	If a potentially hazardous drug-drug combination is prescribed, the prescriber shows knowledge of the hazard.										
4	If the total daily dose is outside the range stated in the BNF or SPC, the prescriber gives a valid reason.										
b Key											
BNF sub-chapter	Valid indications (licensed and unlicensed), as listed in the BNF^a^										
Hypnotics & anxiolytics	Anxiety, insomnia, alcohol dependence, benzodiazepine dependence and allergies.										
Antipsychotics and antimanics	Schizophrenia, psychosis, bipolar disorder, epilepsy, severe aggression or agitation.										
Antidepressants	Depression, anxiety, bipolar disorder, obsessive compulsive disorder and post-traumatic stress disorder.										
CNS Stimulants	Attention Deficit Hyperactivity Disorder										

Note: Denominators vary due to missing data (handled via listwise deletion)

^a^ Note that not all indications are valid for all drugs in each BNF subchapter

Overall, valid (BNF listed) indications for psychotropic drugs were recorded in 65.3 % (63.0–67.5 %) of cases: prescriptions issued to women were slightly more likely to be accompanied by a valid indication than those issued to men (PR 1.18 CI 1.11–1.27; Table 4). The most common invalid indications recorded for antidepressant and antipsychotic prescriptions were low mood (*n* = 94) and personality disorder (*n* = 54), respectively. Other invalid diagnoses commonly recorded included anxiety, insomnia and agitation.

In 93 % of cases overall (93.5 % and 91.4 % for men and women respectively), the total daily dose prescribed was within the range specified in the BNF, thus meeting the PAI indicator. By some margin, the medicine most commonly prescribed at a sub-therapeutic dose was the antipsychotic quetiapine (37 % of cases); indeed, the median prescribed dose across all quetiapine prescriptions was 300 mg (37.5 % of the BNF maximum). One in five (19.4 %) antipsychotic prescriptions were issued at a sub-therapeutic dose, lower than recommended to treat psychoses. Only six cases were identified where the prescribed dose exceeded the maximum recommended dose. Three of these prescriptions were for duloxetine, a type of antidepressant (usually prescribed at 60 mg once daily).

Potentially hazardous drug-drug interactions involving a psychotropic medication, and with no evidence of acknowledgement by the prescriber, were noted in 15.7 % of prescriptions. The most frequently observed interactions were: antipsychotics and antiepileptics (*n* = 33); SSRI and non-steroidal anti-inflammatory drugs (*n* = 31); SSRI and antiepileptics (*n* = 27); and antipsychotics and methadone (*n* = 24). Among those prescribed antipsychotics, hazardous interactions were slightly more common among women than men (PR 1.17 CI 1.01–1.35).

## Discussion

The findings of this study showed a high prevalence of psychotropic prescribing in English prisons, with 47.9 % of women and 16.9 % of men prescribed at least one psychotropic medicine. After adjustment for age, the prevalence of psychotropic prescribing was six times higher among women and four times higher among men, when compared with the general population. Our findings are consistent with previous studies, which have reported frequent psychotropic prescribing among prisoners in England [11, 20], other Western European countries [12–15] and the USA [16, 17]. Whilst psychotropic drugs can help to manage symptoms of mental illness, they have also been linked with addiction, unpleasant side effects, physical health risks and even early mortality [25, 26]. Thus, unnecessary or inappropriate prescribing can carry clinical, as well as cost, implications. It is well-established that prisoners have raised prevalence of mental disorder, three to four times higher than in the general population [2]. Nonetheless, the proportion of prisoners who were prescribed psychotropic medicines in this study, especially among women, appeared to be disproportionately elevated in comparison with the wider community. Furthermore, a third of prescriptions for psychotropic medicines issued in prison were prescribed for undocumented or unapproved indications, not listed in the BNF.

The prevalence of psychotropic prescribing was strikingly high among women in prison; indeed, in the middle age bands (35–54 years), the majority of women were prescribed such medicines. Several factors could be involved. First, a proportion of prescribing will be attributable to the higher prevalence of mental illness seen among women in prison. Second, due to a variety of clinical, social and situational factors, women are said to be differently and disproportionately affected by imprisonment [27]. Third, historically there has been a tendency to over medicalise the social and behavioural problems of women who offend [28]. Set in this wider context, it is possible that medication has now become the default treatment response not only to mental illness, but also challenging behaviours and distress. Greater access to psychological therapies and support for vulnerable individuals in custody could be possible ways to reduce reliance on psychotropic medication.

Prescribing rates for CNS stimulants were much higher among prisoners than primary care patients in the general population. Whilst absolute numbers were small and confidence intervals were wide, this finding does seem to be broadly compatible with previous research, which has indicated that ADHD is disproportionately more common among offenders, both in childhood and in adults [29]. As with other mental illnesses, medicines may be considered an integral part of effective treatment packages for individuals with ADHD in prison, enabling greater engagement with therapeutic and rehabilitative programs. Nonetheless, given that drugs within this therapeutic class have a high propensity for diversion, abuse and dependence, prescribers should continue to exercise caution when prescribing in prisons, especially where there is a history of substance misuse [30].

In this study, it was not uncommon for psychotropic medicines to be prescribed for a wider range of indications than is currently recommended, in particular for low mood (rather than a formal diagnosis of depression) and personality disorder. One possible explanation is that prisons lack the precision to distinguish between, and respond to, mental illness, distress and challenging behaviours, not all of which should require treatment with psychotropic medicines. Such patterns and prescribing practices, whilst arguably concerning, may not necessarily be unique to prisons. Indeed, whilst no drugs are currently licensed for the treatment of personality disorder, one study reported that four fifths of patients with a primary diagnosis of personality disorder under the care of community mental health services received psychotropic medication [31]. Furthermore, we found that a fifth of all antipsychotics were prescribed at sub-therapeutic doses. Very few psychotropic prescriptions exceeded the recommended dose range listed in the BNF. This finding contrasts with a previous systematic review of the literature on prescribing for prisoners which reported concerns with excessive, rather than inadequate, doses [32]. Unfortunately, the majority of the papers included in the review were conducted in hospital settings, rather than prisons, complicating interpretation.

This study benefits from two large samples of people in prison and the general population, thereby increasing the precision of our prevalence estimates, statistical power and generalisability. Except for our own previous work [20], no other studies have made robust, age-adjusted comparisons of prescribing between prisons and the general population; hence, previous studies have probably underestimated any relative differences. Nonetheless, it was not a random sample and not all prisoners and prison types were included (e.g. high secure prisons). Furthermore, CPRD data were based on prescribing data that were approximately two years older than the prison prescribing data that we collected.

Unfortunately, due to the study design, we could not adjust for the prevalence of mental illness. This limits the extent to which we can draw firm conclusions about whether the prescribing of psychotropic medicines is wholly appropriate and proportionate to the level of need. This was a records-based study and limited to information readily available in clinical notes. Clinical records are regarded as a reliable source of information and have the advantage of recording information in a prospective manner, as clinical events occur. Nonetheless, it is possible that certain diagnoses may have not been recorded or were missed when extracting the information, retrospectively. Also, due to differences in data types and collection methods we could not make certain comparisons between patients in prison with those in the general population. In particular, although we were able to assess some aspects of prescribing appropriateness among prisoners, we could not do this for community patients, without gaining more detailed access to their clinical records. Thus we cannot know whether the issues identified here were necessarily unique to prisons. CPRD data did not include the small proportion of psychotropic prescriptions issued in secondary care settings, resulting in an underestimate of prescribing prevalence in the general population. Finally, in focusing on hypnotic, anxiolytic, antipsychotic, antimanic, antidepressant and CNS stimulant medicines, we inevitably missed a proportion of other medicines which may be prescribed to treat mental illness. This included certain antiepileptic drugs, which whilst primarily used to treat epilepsy, may also be used to treat bipolar disorder [21].

## Conclusions

In summary, this study has generated robust prevalence estimates of psychotropic prescribing for a national sample of male and female prisoners, compared with the general population. Psychotropic medicines were prescribed more frequently in prisons, especially among women, and for a wider range of indications than are currently recommended. The results of this study draw attention to disparities and potentially inappropriate prescribing practices in prisons, which are a critical setting for influencing wider public health. The majority of prisoners serve relatively short sentences (less than six months) before being returned to the community. Thus, the treatment of prisoners with mental illness carries implications not only for prescribers in prisons, but also those working with ex-prisoners in the wider community. Further research is needed to understand the reasons why clinicians are prescribing against current guidelines, whether or not this is unique to prisons, and to determine any clinical implications for future mental and physical health. To reduce reliance on medication, prisons may need to develop a more comprehensive array of treatment responses to address mental illness, challenging behaviours and distress.

## Additional file

Additional file 1:
**Table S1**, proportionate denominators for CPRD point prevalence estimates by gender and BNF subchapter. (DOCX 13 kb)

## Abbreviations

BNF: British National Formulary
CNS: Central nervous system
CPRD: Clinical Practice Research Datalink
NHS: National Health Service
PAI: Prescribing appropriate indicators
PR: Prevalence ratio
