# Supplementary material for: Prevalence and appropriateness of psychotropic medication prescribing in a nationally representative cross-sectional survey of male and female prisoners in England
Source: BMC Psychiatry. 2016 Oct 10;16:346. doi: 10.1186/s12888-016-1055-7 (PMC5057241; doi:10.1186/s12888-016-1055-7)
Supplement: Additional file 1: — Table S1, proportionate denominators for CPRD point prevalence estimates by gender and BNF subchapter. (DOCX 13 kb) [file 12888_2016_1055_MOESM1_ESM.docx]

**additional file 1**

Title: Table S1: Proportionate denominators for CPRD point prevalence estimates by gender and BNF subchapter

| **Sample** | **Age group, n** | | | | | | | |
| --- | --- | --- | --- | --- | --- | --- | --- | --- |
|  | 18-24 | 25-34 | 35-44 | 45-54 | 55-64 | 65-74 | 75 + | All |
| **Men** | | | | | | | | |
| Hypnotics and anxiolytics | 18807 | 25008 | 28975 | 28607 | 23897 | 16120 | 11587 | 153000 |
| Antipsychotics and antimanics | 26527 | 35109 | 37090 | 39602 | 33883 | 22157 | 17249 | 211618 |
| Antidepressants | 10036 | 14624 | 17822 | 17057 | 14547 | 9466 | 7819 | 91371 |
| CNS stimulants | 150059 | 227956 | 270532 | 266973 | 223495 | 157767 | 121565 | 1418347 |
| Any | 19478 | 18317 | 20590 | 19782 | 17218 | 11761 | 9625 | 116772 |
|  | | | | | | | | |
| **Women** | | | | | | | | |
| Hypnotics and anxiolytics | 14075 | 23285 | 26683 | 25928 | 23133 | 17051 | 17835 | 147991 |
| Antipsychotics and antimanics | 19958 | 32416 | 38098 | 38462 | 32945 | 24085 | 26554 | 212519 |
| Antidepressants | 7979 | 13866 | 15062 | 15143 | 13513 | 10587 | 12399 | 88548 |
| CNS stimulants | 143783 | 228765 | 261060 | 257306 | 224179 | 168266 | 180489 | 1463848 |
| Any | 10134 | 15973 | 17205 | 17108 | 15622 | 12439 | 14422 | 102904 |

Footnote: For a full explanation of how these denominators were derived, please refer to section <insert page numbers here> of the manuscript.
